# Acupuncture vs usual care for chronic low back pain: a systematic review and meta-analysis of immediate and intermediate effects

**DOI:** 10.1051/sicotj/2025061

**Published:** 2026-02-03

**Authors:** Spyridon Sotiropoulos, Eleftherios Kalafatis, Evaggelos Michalakakos, Andreas Mavrogenis, George Georgoudis

**Affiliations:** 1 Department of Physiotherapy, University of West Attica Athens Greece; 2 Musculoskeletal Physiotherapy Research Laboratory, University of West Attica Athens Greece; 3 1st Department of Anesthesia, Aretaieion University Hospital, National and Kapodistrian University of Athens Athens Greece

**Keywords:** Acupuncture, Chronic low back pain, Usual care, Meta-analysis, Pain

## Abstract

*Introduction*: Chronic low back pain (CLBP) is a leading global cause of disability. Acupuncture is increasingly integrated into its management, yet its standalone effectiveness compared to usual care remains uncertain. This review aimed to assess the immediate (≤2 weeks) and intermediate (2 weeks–6 months) effects of acupuncture versus usual care on pain and disability in adults with CLBP. *Methods*: A systematic review and meta-analysis of randomized controlled trials was conducted, searching MEDLINE, CENTRAL, Scopus, and PEDro through November 2024. Eligible studies compared acupuncture (body, electroacupuncture, scalp) to usual care (physiotherapy, education, medication, and exercise) in adults with CLBP. Outcomes included pain and disability at immediate and intermediate follow-up. Data were pooled using a random-effects model. Risk of bias was assessed with the PEDro scale, and GRADE was used to evaluate evidence certainty. Sensitivity and subgroup analyses were conducted to explore clinical and methodological heterogeneity and test the reliability of findings. *Results*: A total of 2.956 records were identified, and 8 RCTs (*n* = 1,123 participants) were included in this study. Acupuncture significantly reduced pain at both immediate (SMD = –0.73, 95% CI –1.04 to –0.42) and intermediate (SMD = –1.13, 95% CI –1.82 to –0.43) timepoints. Disability also improved at both follow-ups (immediate: SMD = –0.49, 95% CI –0.68 to –0.30 and intermediate: SMD = –0.79, 95% CI –1.18 to –0.41). Sensitivity analyses confirmed effect robustness, especially in electroacupuncture subgroups. Certainty of evidence ranged from low to very low due to risk of bias, inconsistency, and suspected publication bias. *Discussion*: Acupuncture appears more effective than usual care for reducing pain and disability in adults with CLBP, but the certainty of evidence is low, warranting cautious interpretation.

## Introduction

Chronic low back pain (CLBP) affects over half a billion people worldwide and is a leading cause of years lived with disability [1]. It is defined as pain persisting for more than 12 weeks and is associated with functional limitations, psychological distress, and decreased quality of life [2]. CLBP results in substantial healthcare costs, lost productivity, and disability claims, presenting a significant public health challenge [3]. The condition involves physical, psychological, and social factors that complicate its management [4]. Although clinical guidelines recommend a biopsychosocial approach, real-world application and patient outcomes vary considerably [5].

Treatment options for CLBP include pharmacological therapies such as nonsteroidal anti-inflammatory drugs (NSAIDs), muscle relaxants, and opioids, alongside non-pharmacological interventions like therapeutic exercise, mindfulness-based exercises, physical therapy, and cognitive-behavioral therapy [6–8]. While pharmacological treatments are commonly prescribed for symptom relief, evidence supporting their long-term benefits is limited, and opioids carry risks including dependency, overdose, and increased mortality [9]. Non-pharmacological treatments show variable efficacy, and barriers to access and adherence reduce their overall effectiveness [5]. These limitations have led to increased interest in complementary therapies, including acupuncture, which is often integrated into multimodal management plans for CLBP [10].

However, current clinical guidelines differ in their recommendations: the American College of Physicians includes acupuncture among non-pharmacologic treatment options for chronic low back pain [7], whereas the NICE guideline advises against its use for managing low back pain with or without sciatica [11]. Acupuncture has been evaluated in numerous clinical trials and systematic reviews, with evidence suggesting it may reduce pain and improve function in CLBP [10, 12]. However, prior meta-analyses often include heterogeneous comparator groups – including sham, placebo, usual care, or adjunctive therapies – which complicates the isolation of acupuncture’s effect compared to usual clinical management [13, 14]. Additionally, inconsistent definitions of usual care and variability in outcome timing across studies limit the clinical interpretability of previous findings. Existing systematic reviews often pool outcomes from broad and inconsistent follow-up periods, which limits the ability to distinguish acupuncture’s immediate and intermediate effects [15]. Furthermore, many reviews do not consistently exclude sham or adjunct comparators, making it difficult to draw conclusions about acupuncture’s standalone effectiveness.

To address these gaps, this systematic review and meta-analysis focuses exclusively on randomized controlled trials comparing acupuncture alone with well-defined usual care. It applies standardized follow-up categories to separately analyze immediate (within 2 weeks post-treatment) and intermediate (2 weeks to 6 months) effects. Sensitivity and subgroup analyses are conducted to explore clinical and methodological heterogeneity and test the reliability of findings. Although long-term effects were planned for evaluation, no eligible studies with follow-up beyond six months were identified. This review aims to provide a clearer estimate of acupuncture’s immediate and intermediate effects on pain and disability in adults with CLBP, contributing to the evidence to support clinical decision-making and guideline development.

## Materials and methods

This systematic review was conducted in accordance with the PRISMA 2020 guidelines [16]. The protocol was prospectively registered with the International Prospective Register of Systematic Reviews (PROSPERO; CRD42024512458).

### Study design and eligibility criteria

We only included randomized controlled trials (RCTs) comparing acupuncture interventions to usual care in adults with chronic low back pain (CLBP), defined as pain persisting for at least 12 weeks between the lower rib margin and the gluteal fold. Studies were excluded if they were non-randomized, observational, or combined acupuncture with co-interventions applied only to the experimental group. Studies involving serious spinal pathology, recent spinal surgery (<12 months), or primary radicular symptoms were also excluded.

Eligible interventions included body acupuncture, electroacupuncture, dry needling, auricular, and cranial acupuncture. Comparators were restricted to usual care or active treatments such as physiotherapy, medications, and exercise. Studies comparing acupuncture to sham, placebo, passive controls (e.g., waitlist, no treatment), thread embedding, or bee venom acupuncture were excluded to focus on clinically relevant comparisons.

### Outcomes

The primary outcome of this review was considered the pain intensity, assessed using validated related instruments (e.g., VAS, NRS). The secondary outcome was the functional disability, measured with instruments such as the Oswestry Disability Index (ODI), Roland-Morris Disability Questionnaire (RMDQ), or Hannover Functional Ability Questionnaire (HFAQ). Other outcomes (e.g., quality of life, depression, kinesiophobia, physical performance) were extracted where available, but were not pooled due to insufficient data.

### Search strategy and study selection

The databases searched included MEDLINE (via PubMed), Cochrane CENTRAL, Scopus, and PEDro from inception to November 2024. Only studies in the English language were included. Reference lists of included studies and relevant reviews were also screened. Search strategies combined MeSH and free-text terms for “acupuncture”, “chronic low back pain”, and “randomized controlled trial”.

Records were imported into the Rayyan Software [17] for de-duplication and screening. Two reviewers (E.M., E.K.) independently screened titles, abstracts, and full texts. Disagreements were resolved by consensus or a third reviewer (S.S.). A PRISMA 2020 flow diagram summarizes the selection process.

Data were independently extracted by two reviewers using a piloted Excel spreadsheet, initially tested on five studies. Extracted items included study identifiers, sample sizes, participant demographics, intervention and comparator characteristics (e.g., treatment modality, frequency, duration), outcome measures, follow-up timepoints, and summary statistics. When change-from-baseline data were unavailable, immediate post-treatment values were extracted.

### Risk of bias assessment

Risk of bias was assessed independently by two reviewers using the PEDro scale. Discrepancies were resolved through discussion and consensus. Studies were classified as having low (scores 1–3), moderate (4–6), or high (7–10) methodological quality. The PEDro scale evaluates internal validity and statistical reporting, including random allocation, allocation concealment, blinding, and intention-to-treat analysis and is widely accepted and used in the field of physiotherapy [18]. PEDro scores were used to inform both subgroup and sensitivity analyses.

### Outcome timing

Follow-up was categorized based on commonly accepted definitions in the evidence synthesis literature. Immediate-term was defined as assessments conducted within two weeks post-treatment, intermediate-term as those conducted between more than two weeks and up to six months, and long-term follow-up as those beyond six months [19, 20].

### Data synthesis and meta-analysis

Α Meta-analysis of the data was performed using the Review Manager v.5.4 software [21]. Standardized mean differences (SMDs) with 95% confidence intervals (CIs) were calculated for continuous outcomes. A random-effects model was used due to the expected clinical heterogeneity. Heterogeneity was assessed using the I² statistic. Unit-of-analysis issues were addressed following Cochrane recommendations. When pooling was not feasible, a narrative synthesis was used.

Pre-specified sensitivity analyses were conducted to test the consistency of the findings and the sources of heterogeneity. These included: restricting analyses to trials using electroacupuncture (EA) to try and isolate the effect of an almost consistent modality, and excluding statistical outliers identified through visual inspection of forest plots based on extreme effect sizes and wide confidence intervals. Subgroup analyses were conducted based on methodological quality (PEDro score ≥6 vs <6), where data allowed [22–24]. See Supplementary Material 1 for sensitivity analysis forest plots.

### GRADE assessment

The certainty of evidence for each primary outcome – pain and disability at immediate and intermediate-term – was evaluated using the GRADE approach. This involved the assessment of five domains: risk of bias, inconsistency, imprecision, indirectness, and publication bias. The decisions to downgrade were based on prespecified criteria in accordance with GRADE guidelines [25].

### Publication bias

Funnel plots were used to visually assess publication bias. Due to the small number of studies per outcome (<10), no formal tests (e.g., Egger’s test) were performed, in line with Cochrane guidance [23].

### Deviations from PROSPERO

All planned methods were followed, unless otherwise justified. Subgroup analysis by comparator type was not performed due to heterogeneity and low frequency of repeated comparator categories. GRADE assessment and post-hoc sensitivity analyses (e.g., EA-only, outlier exclusion) were added to enhance interpretability and validate the results. The full protocol is available in PROSPERO (CRD42024512458).

## Results

### Study selection

**Figure 1:**
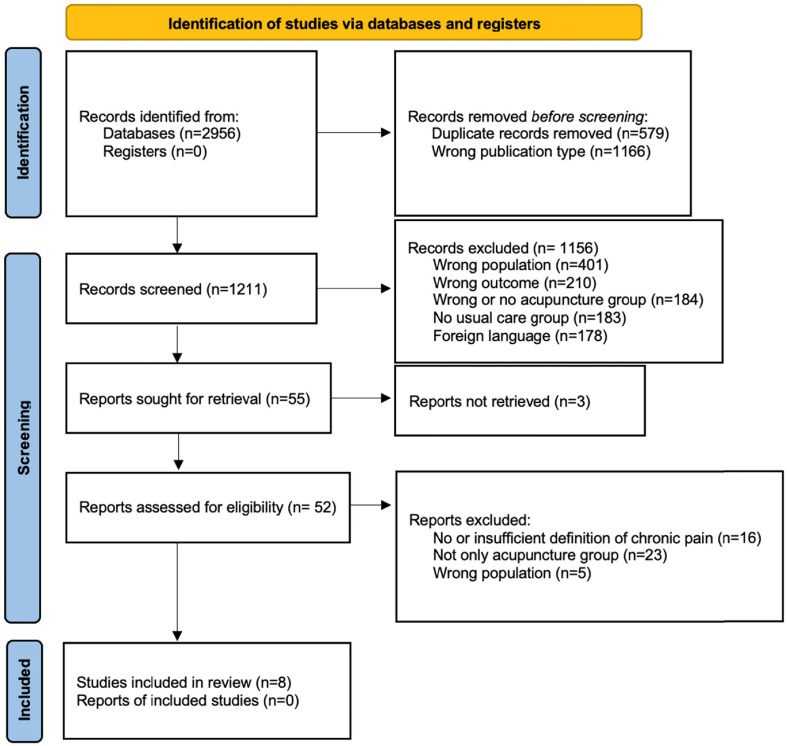
PRISMA 2020 flow diagram for study selection.

A total of 2,956 records were identified through systematic searches of electronic databases. After removing 579 duplicate records and 1,166 records of incorrect publication type (e.g., protocols, editorials), 1,211 unique records remained for title and abstract screening. Of these, 1,156 were excluded due to ineligible population (*n* = 401), outcomes not relevant to this review (*n* = 210), absence of an acupuncture intervention (*n* = 184), absence of a usual care comparator (*n* = 183), or language other than English (*n* = 178). Fifty-five full-text reports were sought for retrieval, with three unavailable. The remaining 52 reports were screened in full; 44 were excluded for reasons including lack of a clear chronic pain definition (*n* = 16), use of mixed or non-isolated acupuncture groups (*n* = 23), and ineligible populations (*n* = 5). Eight randomized controlled trials met all inclusion criteria and were included in the review. Study selection is illustrated in the PRISMA 2020 flow diagram (Figure 1).

### Study characteristics

The eight included randomized controlled trials were conducted between 2007 and 2024 across six countries (Germany, Iran, India, Brazil, China, and Spain), with a total pooled sample of 1,123 participants allocated to acupuncture (*n* = 527) or usual care (*n* = 521). Intervention types varied, though electroacupuncture was the most used modality (6/8 studies), with one trial using auricular acupuncture and another using Yamamoto scalp acupuncture. Comparator interventions reflected standard or routine care in each setting, including pharmacological management (e.g., NSAIDs), physical therapy modalities (e.g., TENS, ultrasound, hot packs), exercise therapy, and health education. Treatment durations ranged from 5 days to 6 weeks, and most studies reported outcomes at both the immediate term and intermediate term. Pain and disability were consistently measured using validated instruments (VAS, ODI, RMDQ), while a minority of studies also assessed quality of life, depression, or inflammatory biomarkers. Adverse events were infrequently reported and were generally mild when described. A detailed overview of the included studies is presented in Table 1.

**Table 1:** Summary and characteristics of the included studies.

Author (year)	Sample size	Age	Experimental	Comparator	Duration	Measured outcomes	Adverse events
Haake et al. (2007) [26]	Exp = 366, *C* = 361, *N* = 727	50.5	Body acupuncture (10 sessions over 5 weeks)	Usual care (physiotherapy, NSAIDs, advice)	5 weeks	Pain: VAS; Disability: FFbHR; Quality of life: SF-36; Functional capacity: Work ability index	Not reported
Zaringhalam et al. (2010) [27]	Exp = 30, *C* = 22, *N* = 52	48.5	Electroacupuncture 2×/week	NSAIDs (piroxicam)	4 weeks	Pain: VAS; Disability: RMDQ; Quality of life: SF-36; Biomarkers: TNF-α	No adverse events reported
Shankar et al. (2011) [28]	Exp = 30, *C* = 30, *N* = 60	45.3	Electroacupuncture daily (5 days/week)	Physiotherapy + analgesics	4 weeks	Pain: VAS; Disability: RMDQ; Functional status	No adverse events reported
Camilotti et al. (2015) [29]	Exp = 15, *C* = 15, *N* = 30	51.2	Yamamoto scalp acupuncture	Aquatic physiotherapy	12 weeks	Pain: VAS; Quality of life: WHOQOL-BREF	Not reported
Meng et al. (2022) [30]	Exp = 30, *C* = 30, *N* = 60	45.9	Electroacupuncture 2×/week	Exercise therapy	8 weeks	Pain: VAS; Disability: ODI; Depression: HAMD	Minor: bruising, subcutaneous bleeding
Cheng et al. (2023) [31]	Exp = 30, *C* = 30, *N* = 60	47.3	Electroacupuncture 3×/week	Health education	4 weeks	Pain: VAS; Disability: ODI; Quality of life: SF-36	No adverse events reported
Moein Jamali Dastjerdi et al. (2024) [32]	Exp = 30, *C* = 31, *N* = 61	41.6	Electroacupuncture daily (10 sessions)	NSAIDs (celecoxib) + exercise	2 weeks	Pain: VAS; Disability: ODI; Depression: BDI	Not reported
Lara-Palomo et al. (2024) [33]	Exp = 32, *C* = 32, *N* = 64	43.1	Electroacupuncture 2×/week	Physiotherapy	6 weeks	Pain: VAS; Disability: ODI	No adverse events reported

*Abbreviations: Exp = Experimental; C = Comparator; VAS = Visual Analog Scale; FFbHR = Hannover Functional Ability Questionnaire; SF-36 = 36-Item Short Form Health Survey; WAI = Work Ability Index; RMDQ = Roland-Morris Disability Questionnaire; TNF-α = Tumor Necrosis Factor-alpha; WHOQOL-BREF = World Health Organization Quality of Life – BREF; ODI = Oswestry Disability Index; HAMD = Hamilton Depression Rating Scale; BDI = Beck Depression Inventory.

### Risk of bias

Methodological quality was moderate to high across included studies, with PEDro scores ranging from 4 to 8 out of 10. Three trials scored 8/10, reflecting strong adherence to key methodological criteria, including randomization, concealed allocation, and assessor blinding [26, 30, 33]. Studies were categorized as low (1–3), moderate (4–6), or high (7–10) quality, consistent with previously established thresholds for interpreting PEDro scores [18]. One study scored 4/10, primarily due to lack of blinding, incomplete follow-up, and absence of intention-to-treat analysis [29]. Blinding of subjects and therapists was generally absent, which is common in acupuncture trials. Sensitivity and subgroup analyses were conducted to explore the influence of methodological quality on pooled effects (Supplementary Material 1). Full PEDro ratings are presented in Supplementary Material 3.

### Narrative synthesis of outcomes

All eight included studies reported outcomes on pain and disability, which were the primary focus of this review. Pain was measured using the Visual Analogue Scale (VAS) or Numeric Pain Rating Scale (NPRS) in all trials [26–33], with all showing significant post-treatment reductions in the acupuncture groups. For example, Zaringhalam et al. [27] reported significantly greater VAS reductions in acupuncture and acupuncture + baclofen groups compared to baclofen alone. Camilotti et al. [29] found VAS scores decreased significantly in the YNSA and Tai Chi groups relative to the control (*p* < 0.05). Meng et al. [30] showed a ≥2-point NRS reduction in 65% of participants after electroacupuncture; and Lara-Palomo et al. [33] observed sustained pain reductions at both 6-week and 2-month follow-up. Disability was assessed using the Oswestry Disability Index (ODI), Roland-Morris Disability Questionnaire (RMDQ), or Hannover Functional Ability Questionnaire (FFbHR). Cheng et al. [31] found greater ODI improvements in the electroacupuncture and lumbar-pelvic training group compared to either modality alone; Lara-Palomo et al. [33] found significant ODI and RMDQ reductions in the dry needling group, and Haake et al. [26] reported functional gains in the acupuncture arm using the FFbHR. All studies reported immediate-term outcomes, while four trials [26, 27, 32, 33] included intermediate-term investigation of outcomes, showing maintained benefits in pain and disability. Acupuncture generally resulted in greater reductions of pain and disability compared to usual care, though the magnitude and consistency of effect varied.

Secondary outcomes were reported in a subset of studies. Quality of life was assessed in three trials using validated instruments (SF-36 or WHOQOL-BREF). Lara-Palomo et al. [33] reported significant improvements in the SF-36 domains of physical functioning, vitality, and sleep efficacy in the electrical dry needling group (*p* < 0.012). Meng et al. [30] found improvements in the physical and psychological domains of the WHOQOL-BREF following electroacupuncture. Camilotti et al. [29] observed improvements in the psychological and physical health domains on the WHOQOL-BREF in participants receiving Yamamoto scalp acupuncture and Ai Chi. Shankar et al. [28] reported better autonomic status post-treatment. Depression scores (HAMD, BDI) were reported in two studies [28, 30], both showing within-group reductions after acupuncture. Functional outcomes, including trunk muscle endurance and lumbar mobility, improved in the dry needling group of Lara-Palomo et al. [33]. One study measured changes in inflammatory status and found a significant reduction in serum TNF-α levels post-treatment with electroacupuncture [30]. Due to variation in instruments and reporting formats, these secondary outcomes were not included in the meta-analysis but they are presented narratively to reflect the broader therapeutic effects of acupuncture. Meng et al. [30] study was excluded from the meta-analysis due to incomplete reporting of group-level means and standard deviations for pain and disability outcomes, preventing reliable data extraction.

### Meta-analysis results

#### Pain (immediate post-treatment)

Seven RCTs (*n* = 1,048) comparing acupuncture to usual care for immediate-term pain were included in the meta-analysis [25–28, 30–32]. The pooled analysis showed a significant effect favoring acupuncture (SMD = –0.73; 95% CI –1.04 to –0.42; *p* < 0.00001; *I*² = 68%) (Table 2).

**Table 2:**
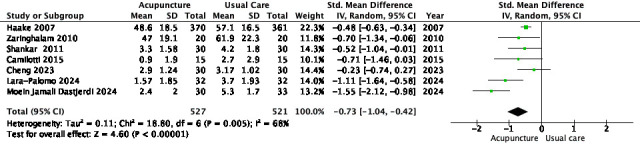
Meta-analysis forest plot of acupuncture versus usual care for immediate-term differences in pain.

A sensitivity analysis restricted to trials using electroacupuncture [27, 28, 31–33] produced a similar effect (SMD = –0.82; 95% CI –1.28 to –0.36; *I*² = 71%). A second sensitivity analysis removing three statistical outliers – [29, 32, 33] – identified by their extreme effect sizes and wide confidence intervals, identified by their extreme effect sizes and wide confidence intervals, yielded a significant pooled effect (SMD = –0.48; 95% CI –0.61 to –0.35) with heterogeneity reduced to 0%.

Subgroup analysis by methodological quality (PEDro score ≥6 vs <6) showed a stronger effect in higher-quality trials (SMD = –0.93 vs –0.44), although the interaction was not statistically significant (*p* = 0.12).

#### Disability (immediate post-treatment)

Six RCTs (*n* = 988) comparing acupuncture to usual care for immediate-term disability were included in the meta-analysis [26, 27, 29, 31–33]. The pooled analysis demonstrated a moderate, statistically significant effect favoring acupuncture (SMD = –0.49; 95% CI –0.68 to –0.30; *p* < 0.00001; *I*² = 20%) (Table 3).

**Table 3:**
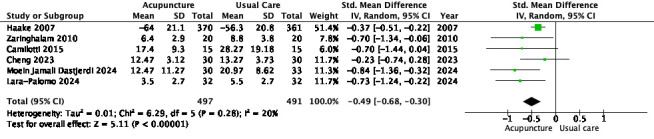
Meta-analysis forest plot of acupuncture versus usual care for immediate-term differences in disability.

Sensitivity analysis limited to electroacupuncture trials showed a slightly larger effect size (SMD = –0.62; 95% CI –0.89 to –0.34; *I*² = 7%), indicating consistency across modality-specific interventions [27, 31–33]. A second sensitivity analysis excluded two statistical outliers – [27, 29] – based on their disproportionate contribution to heterogeneity and wide confidence intervals. The effect remained stable (SMD = –0.48; 95% CI –0.73 to –0.24), with *I*² rising to 40%.

Subgroup analysis based on methodological quality (PEDro score ≥6 vs <6) revealed a slightly stronger effect in higher-quality trials (SMD = –0.57 vs –0.39), although the interaction test was not statistically significant (*p* = 0.49).

#### Pain (intermediate term follow up)

Four RCTs (*n* = 908) comparing acupuncture to usual care for intermediate-term pain outcomes were included in the meta-analysis [26, 27, 32, 33]. The pooled analysis revealed a large, statistically significant effect favoring acupuncture (SMD = –1.13; 95% CI –1.82 to –0.43; *p* = 0.001; *I*² = 89%) (Table 4).

**Table 4:**
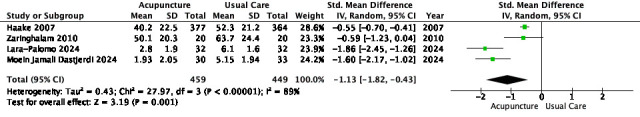
Meta-analysis forest plot of acupuncture versus usual care for intermediate-term follow-up differences in pain.

Sensitivity analysis excluding an outlier based on extreme effect size and narrow confidence interval reduced heterogeneity while maintaining a significant effect (SMD = –0.89; 95% CI –1.52 to –0.25; *I*² = 83%) [33]. A second sensitivity analysis excluding the largest study with non-typical baseline values and comparator variation yielded a comparable result (SMD = –1.36; 95% CI –2.09 to –0.63; *I*² = 78%) [26].

An electroacupuncture-only sensitivity analysis preserved a strong treatment effect (SMD = –1.36; 95% CI –2.09 to –0.63; *I*² = 78%), supporting consistency across stimulation modalities [27, 32, 33].

#### Disability (intermediate term follow-up)

Four RCTs (*n* = 908) assessed intermediate-term disability outcomes comparing acupuncture with usual care [26, 27, 32, 33]. Meta-analysis indicated a moderate-to-large, statistically significant effect favoring acupuncture (SMD = –0.79; 95% CI –1.18 to –0.41; *p* < 0.0001; *I*² = 68%) (Table 5).

**Table 5:**
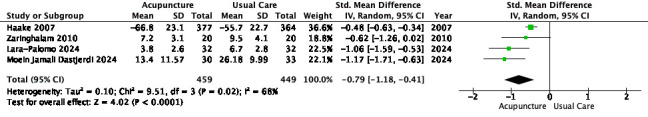
Meta-analysis forest plot of acupuncture versus usual care for intermediate-term differences in disability.

During the sensitivity analysis, the exclusion of a statistically significant outlier with a high effect size and low variance reduced heterogeneity (SMD = –0.66; 95% CI –1.02 to –0.31; *I*² = 54%) while preserving statistical significance [32]. A more conservative model yielded a comparable pooled estimate with low heterogeneity (SMD = –0.88; 95% CI –1.30 to –0.45; *I*² = 8%) [27, 32].

In the electroacupuncture-only sensitivity analysis, the pooled effect remained statistically significant and homogenous (SMD = –0.99; 95% CI –1.31 to –0.66; *I*² = 0%) [27, 32, 33].

### Adverse events

Adverse events were reported in only one study, which documented minor effects such as bruising in the electroacupuncture group [30]. No serious adverse events were observed in any of the included trials.

### Publication bias

Formal assessment of publication bias using Egger’s test or the Trim-and-Fill method was not conducted due to the limited number of included studies per outcome (*n* < 10), in accordance with Cochrane guidelines [20].

Visual inspection of funnel plots for all primary outcomes revealed varying degrees of asymmetry, suggesting potential small-study effects (Supplementary Material 4). For immediate pain outcomes, the funnel plot showed moderate asymmetry, with several smaller studies favoring acupuncture. The intermediate pain funnel plot displayed more pronounced asymmetry, with study effects clustering on the left, indicating possible publication bias toward positive findings. See Supplementary Material 5.

Similarly, the funnel plot for immediate post-treatment disability showed slight asymmetry, while subgroup plots stratified by PEDro score suggested more balanced distributions, especially among higher-quality trials. The intermediate disability funnel plot showed mild asymmetry, with electroacupuncture-only sensitivity plots appearing more consistent, though still limited by the small sample size.

### GRADE evidence summary

The certainty of evidence was assessed using the GRADE approach. Two outcomes were rated as having low-certainty evidence: immediate pain reduction and immediate disability reduction, due to downgrades for risk of bias, inconsistency, and suspected publication bias. The remaining two outcomes, intermediate-term pain and disability reduction, were rated as **very low certainty**, reflecting additional concerns about imprecision. GRADE results are summarized in Table 6 and detailed in Supplementary Material 2.

**Table 6:** Summary of findings (SoF) table.

Outcome	No. of studies	Participants (total)	Effect (SMD, 95% CI)	Certainty (GRADE)	Reasons for downgrading
Pain – immediate	7	1048	–0.73 [–1.04, –0.42]	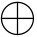 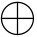 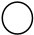 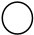 Low	Risk of bias; publication bias
Disability – immediate	6	988	–0.49 [–0.68, –0.30]	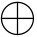 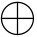 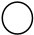 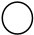 Low	Risk of bias; publication bias
Pain – intermediate-term follow-up	4	908	–1.13 [–1.82, –0.43]	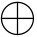 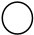 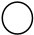 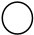 Very Low	Risk of bias, inconsistency, publication bias
Disability – intermediate-term follow-up	4	908	–0.79 [–1.18, –0.41]	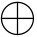 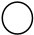 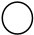 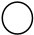 Very Low	Risk of bias; inconsistency; imprecision; publication bias

Summary of pooled effects for the four primary outcomes comparing acupuncture to usual care in adults with chronic low back pain. Standardized mean differences (SMDs) with 95% confidence intervals (CIs) are presented. Certainty of evidence was assessed using the GRADE approach. Reasons for downgrading are detailed for each outcome.

## Discussion

This systematic review and meta-analysis aimed to evaluate the immediate and intermediate effects of acupuncture compared to usual care in adults with chronic low back pain. Our results demonstrate that acupuncture provides statistically significant and clinically meaningful reductions in pain and disability at both immediate and intermediate follow-up. In contrast, the usual care showed smaller and often non-significant improvements. These findings clarify acupuncture’s standalone effectiveness relative to usual care, addressing uncertainties from prior reviews with heterogeneous comparators.

Our meta-analysis demonstrated statistically significant reductions in pain and disability with acupuncture compared to usual care across immediate and intermediate timepoints. Specifically, immediate term pain was reduced with a moderate effect size, while immediate disability showed a smaller but significant effect of improvement. Intermediate term outcome analysis revealed larger effect sizes for both pain and disability. The certainty of evidence ranged from moderate for immediate pain relief to very low for intermediate disability outcomes, primarily due to risk of bias, heterogeneity, and imprecision. Therefore, while acupuncture appears effective compared to usual care, these findings should be interpreted with caution, considering the methodological limitations and the clinical context.

Our findings are generally consistent with previous meta-analyses, though effect sizes varied depending on inclusion criteria and comparator definitions. Mu et al. (2020) reported smaller pooled effects (SMD –0.30 to –0.50) when comparing acupuncture with usual care or no treatment, likely reflecting their inclusion of sham and heterogeneous comparators [12]. Giovanardi et al. (2023) found moderate-to-large benefits for pain (SMD = –0.68) and disability (SMD = –0.57), comparable to our immediate-term results [14]. Similarly, Asano et al. (2022) reported moderate effects when acupuncture was used adjunctively with standard therapy [13]. Differences in comparator definitions, participant characteristics, and outcome timing likely explain the variability in pooled estimates. The larger intermediate-term effects observed in our review suggest that acupuncture’s standalone benefits may be underestimated in prior reviews with broader comparator groups [10, 20, 34, 35].

Sensitivity analyses confirmed the stability of acupuncture’s effects on pain and disability, with consistent effect sizes after restricting to electroacupuncture and excluding outliers, which reduced heterogeneity. Subgroup analyses by study quality showed larger, though non-significant, effects in higher-quality trials, supporting the reliability of the findings across methodological variations. These results indicate that the benefits of acupuncture for chronic low back pain are robust and generalizable across different acupuncture techniques and study designs.

The findings of this review suggest that acupuncture offers a valuable add-on or an alternative to usual care for adults with chronic low back pain, providing meaningful reductions in pain and disability at both immediate and intermediate timepoints [7, 10]. Clinicians could consider acupuncture as a non-pharmacological option for chronic LBP patients, particularly for those who may need to avoid long-term use of analgesics, NSAIDs, or opioids [6, 9] . The small number of reported adverse events in this study aligns with existing evidence supporting acupuncture’s favorable safety profile [36]. Considering both the observed effect sizes and its well-documented tolerability, acupuncture may appear appropriate for patients seeking minimally invasive, low-risk treatment options. Our observed effect sizes for pain reduction (e.g., SMD = –0.73, immediate term) suggest a moderate to large clinical effect, consistent with meaningful patient improvement in chronic low back pain [37–39]. However, due to heterogeneity in study populations and interventions, further research is needed to identify specific subgroups that may enjoy the greatest benefits (e.g., pain severity, duration, or comorbidities), as factors such as employment status and health behaviors have been shown to influence disability outcomes in chronic low back pain [5, 40]. In clinical practice, integrating acupuncture within multidisciplinary pain management strategies may optimize patient outcomes while minimizing reliance on medications with higher risk profiles [7, 9].

This systematic review used a targeted comparator approach, including only trials comparing acupuncture with usual care. This approach improves clinical relevance by excluding studies with diverse comparators, such as sham or additional treatments. [13, 14]. The methodology included predefined outcome timepoints aligned with Cochrane recommendations and incorporated sensitivity and subgroup analyses alongside formal GRADE assessments to evaluate evidence certainty [20, 25]. Limitations of this review include a moderate risk of bias in the employed studies, especially related to blinding and allocation concealment. These issues may affect the accuracy of the results and could lead to an overestimation of the treatment effects. [23, 41]. The relatively small number of trials and participant samples may have reduced the statistical power and increased the susceptibility to random error [34]. Importantly, three studies were excluded from the review because their full texts could not be obtained. This limitation introduces a level of uncertainty regarding the appropriateness of their exclusion, since access to the complete reports may have revealed eligibility for inclusion. Funnel plot asymmetry suggested potential publication bias and small-study effects; however, formal statistical tests such as Egger’s regression were not conducted due to the limited number of studies per outcome, consistent with recommended practice [42, 43]. Visual inspection alone is insufficient to conclusively assess publication bias, and small-study effects may exaggerate pooled estimates [44]. Furthermore, restricting inclusion criteria to English-language publications may have introduced language bias, limiting the comprehensiveness and generalizability of findings to broader populations [45, 46]. Variability in acupuncture protocols across studies and the lack of accurately defining low back pain subpopulations may further have impacted the applicability of results [5, 10]. These factors contributed to the downgrading of the evidence certainty, particularly for intermediate disability outcomes, which were rated very low [25]. Recognizing the strengths and limitations of this review is essential for accurate interpretation and application of the findings in clinical and research settings.

This systematic review and meta-analysis found that acupuncture, compared to usual care, was associated with greater reductions in pain and disability among adults with chronic low back pain at both immediate and intermediate follow-up. The effects were consistent across studies and particularly notable in trials assessing the use of electroacupuncture. However, the overall certainty of evidence was rated low to very low, primarily due to risks of bias, inconsistency in results, and suspected publication bias. These limitations restrict the confidence with which clinical recommendations can be made. In clinical practice, these findings support the consideration of acupuncture as a non-pharmacological component of multidisciplinary management for chronic low back pain, particularly for patients seeking to reduce or avoid long-term analgesic use. Future research should focus on evaluating the sustainability of treatment effects through long-term follow-up and assessing cost-effectiveness in real-world care settings to determine the practical value of integrating acupuncture into routine clinical practice.

## Data Availability

All data used in this meta-analysis were extracted from previously published studies. Summary data, forest plots, and characteristics of included trials are presented in the main text and supplementary materials.

## Supplementary material

Supplementary Material 1: Forest Plots of Sensitivity and Subgroup Analyses. Supplementary Material 2: GRADE Summary Tables for Pain and Disability Outcomes. Supplementary Material 3: PEDro scores by Study and Methodological Domain. Supplementary Material 4: Funnel Plots for publication Bias Assessment. Supplementary Material 5: GRADE PRISMA 2020 CHECKLIST. The supplementary material of this article is available at https://sicotj.edpsciences.org/10.1051/sicotj/2025061/olm.
