# Supplementary material for: Acupuncture vs usual care for chronic low back pain: a systematic review and meta-analysis of immediate and intermediate effects
Source: SICOT J. 2026 Feb 3;12:7. doi: 10.1051/sicotj/2025061 (PMC12867475; doi:10.1051/sicotj/2025061)
Supplement: Supplementary file 1 — Supplementary Material 1: Forest Plots of Sensitivity and Subgroup Analyses. Supplementary Material 2: GRADE Summary Tables for Pain and Disability Outcomes. Supplementary Material 3: PEDro scores by Study and Methodological Domain. Supplementary Material 4: Funnel Plots for publication Bias Assessment. Supplementary Material 5: GRADE PRISMA 2020 CHECKLIST. [file sicotj-12-7-s1.zip › sicotj250093-1-olm/Supplementary Material Acup vs Usual Care.pdf]

Supplementary Material 1: Forest Plots of Sensitivity and Subgroup Analyses

Study or Subgroup	Acupuncture Mean	Acupuncture SD	Acupuncture Total	Usual Care Mean	Usual Care SD	Usual Care Total	Weight	Std. Mean Difference IV, Random, 95% CI	Year
Haake 2007	48.6	18.5	370	57.1	16.5	361	31.2%	−0.48 [−0.63, −0.34]	2007
Zaringhalam 2010	47	19.1	20	61.9	22.3	20	21.6%	−0.70 [−1.34, −0.06]	2010
Shankar 2011	3.3	1.58	30	4.2	1.8	30	0.0%	−0.52 [−1.04, −0.01]	2011
Camilotti 2015	0.9	1.9	15	2.7	2.9	15	0.0%	−0.71 [−1.46, 0.03]	2015
Cheng 2023	2.9	1.24	30	3.17	1.02	30	0.0%	−0.23 [−0.74, 0.27]	2023
Lara−Palomo 2024	1.57	1.85	32	3.7	1.93	32	24.1%	−1.11 [−1.64, −0.58]	2024
Moein Jamali Dastjerdi 2024	2.4	2	30	5.3	1.7	33	23.2%	−1.55 [−2.12, −0.98]	2024
Total (95% CI)			452			446	100.0%	−0.93 [−1.45, −0.41]	

Heterogeneity: $Tau^2 = 0.22$; $Chi^2 = 16.83$, df = 3 ($P = 0.0008$); $I^2 = 82\%$
Test for overall effect: $Z = 3.49$ ($P = 0.0005$)

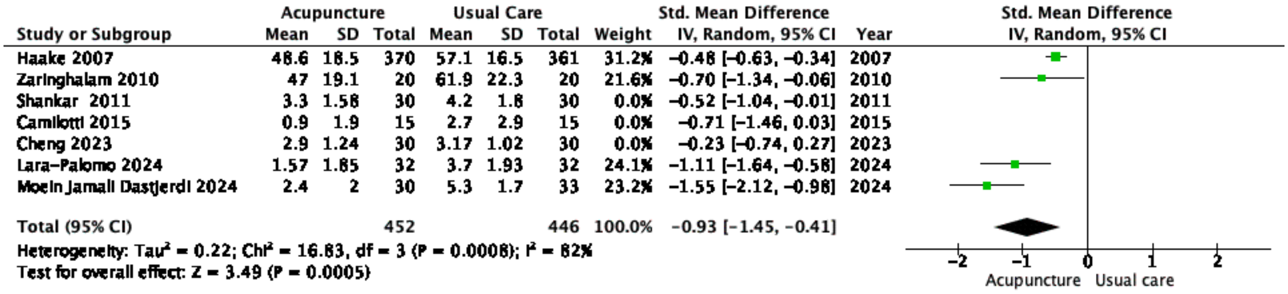

Figure S1. Sensitivity analysis for immediate post-treatment pain comparing acupuncture versus usual care, limited to trials with

PEDro score >6. Standardized mean differences (SMDs) and 95% confidence intervals (CIs) were calculated using a random-effects

model.

Study or Subgroup	Acupuncture Mean	Acupuncture SD	Acupuncture Total	Usual Care Mean	Usual Care SD	Usual Care Total	Weight	Std. Mean Difference IV, Random, 95% CI	Year
Haake 2007	48.6	18.5	370	57.1	16.5	361	0.0%	−0.48 [−0.63, −0.34]	2007
Zaringhalam 2010	47	19.1	20	61.9	22.3	20	18.2%	−0.70 [−1.34, −0.06]	2010
Shankar 2011	3.3	1.58	30	4.2	1.8	30	20.8%	−0.52 [−1.04, −0.01]	2011
Camilotti 2015	0.9	1.9	15	2.7	2.9	15	0.0%	−0.71 [−1.46, 0.03]	2015
Cheng 2023	2.9	1.24	30	3.17	1.02	30	20.9%	−0.23 [−0.74, 0.27]	2023
Lara−Palomo 2024	1.57	1.85	32	3.7	1.93	32	20.5%	−1.11 [−1.64, −0.58]	2024
Moein Jamali Dastjerdi 2024	2.4	2	30	5.3	1.7	33	19.7%	−1.55 [−2.12, −0.98]	2024
Total (95% CI)			142			145	100.0%	−0.82 [−1.28, −0.36]	

Heterogeneity: $Tau^2 = 0.19$; $Chi^2 = 13.98$, df = 4 ($P = 0.007$); $I^2 = 71\%$
Test for overall effect: $Z = 3.49$ ($P = 0.0005$)

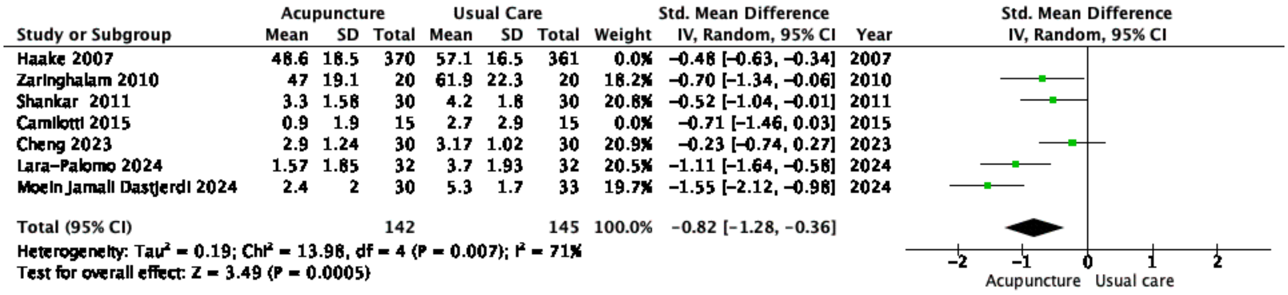

Figure S2. Sensitivity analysis for immediate post-treatment pain comparing electroacupuncture versus usual care. Analysis restricted to trials using electroacupuncture (EA) only. Results are presented as standardized mean differences (SMDs) with 95% confidence intervals (CIs), using a random-effects model.

Study or Subgroup	Acupuncture Mean	Acupuncture SD	Acupuncture Total	Usual Care Mean	Usual Care SD	Usual Care Total	Weight	Std. Mean Difference IV, Random, 95% CI	Year
Haake 2007	48.6	18.5	370	57.1	16.5	361	82.1%	−0.48 [−0.63, −0.34]	2007
Zaringhalam 2010	47	19.1	20	61.9	22.3	20	4.3%	−0.70 [−1.34, −0.06]	2010
Shankar 2011	3.3	1.58	30	4.2	1.8	30	6.7%	−0.52 [−1.04, −0.01]	2011
Camilotti 2015	0.9	1.9	15	2.7	2.9	15	0.0%	−0.71 [−1.46, 0.03]	2015
Cheng 2023	2.9	1.24	30	3.17	1.02	30	6.9%	−0.23 [−0.74, 0.27]	2023
Lara−Palomo 2024	1.57	1.85	32	3.7	1.93	32	0.0%	−1.11 [−1.64, −0.58]	2024
Moein Jamali Dastjerdi 2024	2.4	2	30	5.3	1.7	33	0.0%	−1.55 [−2.12, −0.98]	2024
Total (95% CI)			450			441	100.0%	−0.48 [−0.61, −0.35]	

Heterogeneity: $Tau^2 = 0.00$; $Chi^2 = 1.39$, df = 3 ($P = 0.71$); $I^2 = 0\%$
Test for overall effect: $Z = 7.04$ ($P < 0.00001$)

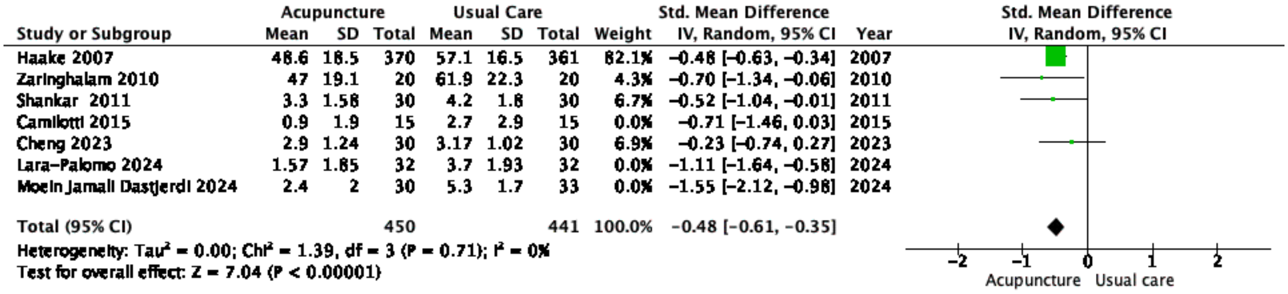

Figure S3. Sensitivity analysis for immediate post-treatment pain comparing acupuncture versus usual care, excluding three statistical outliers. Camilotti 2015, Lara-Palomo 2024, and Moein Jamali Dastjerdi 2024 were excluded based on extreme standardized mean differences and wide confidence intervals. Results show a stable treatment effect and reduced heterogeneity ($I^2 = 0\%$).

Study or Subgroup	Acupuncture Mean	Acupuncture SD	Acupuncture Total	Usual Care Mean	Usual Care SD	Usual Care Total	Weight	Std. Mean Difference IV, Random, 95% CI	Year
**1.5.1 Pain Immediate Low Score Pedro**									
Shankar 2011	3.3	1.58	30	4.2	1.8	30	14.3%	−0.52 [−1.04, −0.01]	2011
Camilotti 2015	0.9	1.9	15	2.7	2.9	15	10.1%	−0.71 [−1.46, 0.03]	2015
Cheng 2023	2.9	1.24	30	3.17	1.02	30	14.4%	−0.23 [−0.74, 0.27]	2023
Subtotal (95% CI)			75			75	38.8%	−0.44 [−0.77, −0.12]	
Heterogeneity: Tau² = 0.00; Chi² = 1.26, df = 2 (P = 0.53); I² = 0%									
Test for overall effect: Z = 2.67 (P = 0.008)									
**1.5.2 Pain Immediate High Score Pedro**									
Haake 2007	48.6	18.5	370	57.1	16.5	361	22.3%	−0.48 [−0.63, −0.34]	2007
Zaringhalam 2010	47	19.1	20	61.9	22.3	20	11.8%	−0.70 [−1.34, −0.06]	2010
Lara-Palomo 2024	1.57	1.85	32	3.7	1.93	32	14.0%	−1.11 [−1.64, −0.58]	2024
Moein Jamali Dastjerdi 2024	2.4	2	30	5.3	1.7	33	13.2%	−1.55 [−2.12, −0.98]	2024
Subtotal (95% CI)			452			446	61.2%	−0.93 [−1.45, −0.41]	
Heterogeneity: Tau² = 0.22; Chi² = 16.83, df = 3 (P = 0.0008); I² = 82%									
Test for overall effect: Z = 3.49 (P = 0.0005)									
Total (95% CI)			527			521	100.0%	−0.73 [−1.04, −0.42]	
Heterogeneity: Tau² = 0.11; Chi² = 18.80, df = 6 (P = 0.005); I² = 68%									
Test for overall effect: Z = 4.60 (P < 0.00001)									
Test for subgroup differences: Chi² = 2.41, df = 1 (P = 0.12), I² = 58.6%									

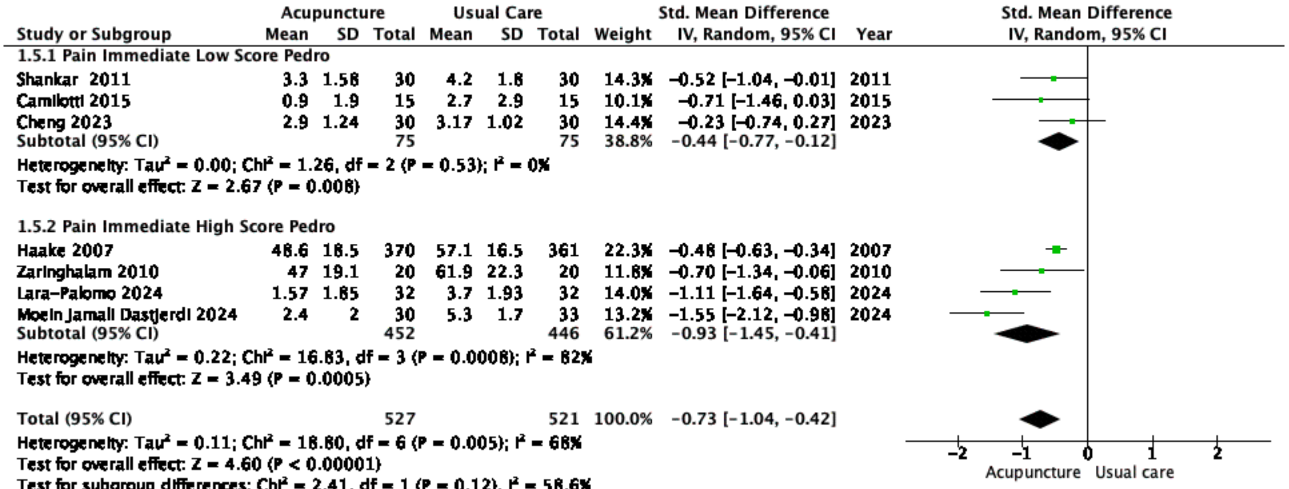

Figure S4. Subgroup analysis for immediate post-treatment pain comparing acupuncture versus usual care, stratified by PEDro score.

Trials were grouped by methodological quality (PEDro <6 vs ≥6). Acupuncture showed significant benefits in both subgroups, with larger effect sizes in higher-quality studies. No statistically significant subgroup interaction was detected (p = 0.12).

Study or Subgroup	Acupuncture Mean	Acupuncture SD	Acupuncture Total	Usual Care Mean	Usual Care SD	Usual Care Total	Weight	Std. Mean Difference IV, Random, 95% CI	Year
Haake 2007	−64	21.1	370	−56.3	20.8	361	0.0%	−0.37 [−0.51, −0.22]	2007
Zaringhalam 2010	6.4	2.9	20	8.8	3.8	20	17.9%	−0.70 [−1.34, −0.06]	2010
Camilotti 2015	17.4	9.3	15	28.27	19.18	15	0.0%	−0.70 [−1.44, 0.04]	2015
Cheng 2023	12.47	3.12	30	13.27	3.73	30	27.7%	−0.23 [−0.74, 0.28]	2023
Moein Jamali Dastjerdi 2024	12.47	11.27	30	20.97	8.62	33	26.7%	−0.84 [−1.36, −0.32]	2024
Lara-Palomo 2024	3.5	2.7	32	5.5	2.7	32	27.7%	−0.73 [−1.24, −0.22]	2024
Total (95% CI)			112			115	100.0%	−0.62 [−0.89, −0.34]	
Heterogeneity: Tau² = 0.01; Chi² = 3.22, df = 3 (P = 0.36); I² = 7%									
Test for overall effect: Z = 4.35 (P < 0.0001)									

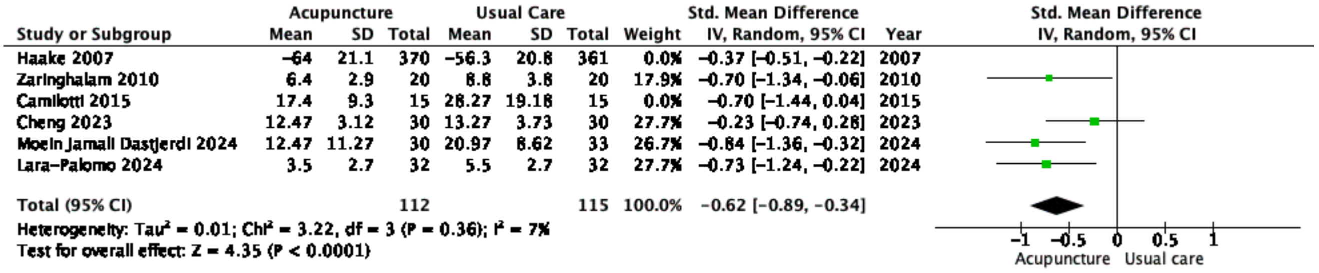

Figure S5. Sensitivity analysis for immediate post-treatment disability comparing electroacupuncture versus usual care.

Only trials using electroacupuncture were included. The pooled analysis demonstrated a moderate effect favoring acupuncture (SMD = –0.62; 95% CI –0.89 to –0.34) with low heterogeneity (I^2 = 7%).

Study or Subgroup	Acupuncture Mean	Acupuncture SD	Acupuncture Total	Usual Care Mean	Usual Care SD	Usual Care Total	Weight	Std. Mean Difference IV, Random, 95% CI	Year
Haake 2007	−64	21.1	370	−56.3	20.8	361	49.5%	−0.37 [−0.51, −0.22]	2007
Zaringhalam 2010	6.4	2.9	20	8.8	3.8	20	0.0%	−0.70 [−1.34, −0.06]	2010
Camilotti 2015	17.4	9.3	15	28.27	19.18	15	0.0%	−0.70 [−1.44, 0.04]	2015
Cheng 2023	12.47	3.12	30	13.27	3.73	30	17.0%	−0.23 [−0.74, 0.28]	2023
Moein Jamali Dastjerdi 2024	12.47	11.27	30	20.97	8.62	33	16.5%	−0.84 [−1.36, −0.32]	2024
Lara-Palomo 2024	3.5	2.7	32	5.5	2.7	32	17.0%	−0.73 [−1.24, −0.22]	2024
Total (95% CI)			462			456	100.0%	−0.48 [−0.73, −0.24]	
Heterogeneity: Tau² = 0.03; Chi² = 5.04, df = 3 (P = 0.17); I² = 40%									
Test for overall effect: Z = 3.84 (P = 0.0001)									

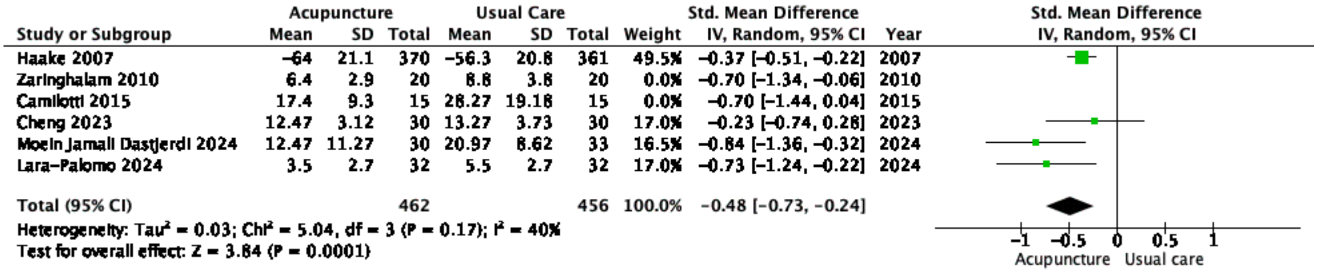

Figure S6. Sensitivity analysis for immediate post-treatment disability comparing acupuncture versus usual care, excluding two statistical outliers.

Zaringhalam 2010 and Camilotti 2015 were excluded based on disproportionate effect sizes and contribution to heterogeneity. Results remained statistically significant with reduced heterogeneity (I^2 = 40%).

Study or Subgroup	Acupuncture Mean	Acupuncture SD	Acupuncture Total	Usual Care Mean	Usual Care SD	Usual Care Total	Weight	Std. Mean Difference IV, Random, 95% CI	Year
**1.6.1 Disability Immediate Subgroup Low Score**									
Camilotti 2015	17.4	9.3	15	28.27	19.18	15	6.0%	−0.70 [−1.44, 0.04]	2015
Cheng 2023	12.47	3.12	30	13.27	3.73	30	11.7%	−0.23 [−0.74, 0.28]	2023
Subtotal (95% CI)			45			45	17.7%	−0.39 [−0.82, 0.05]	
Heterogeneity: Tau² = 0.01; Chi² = 1.06, df = 1 (P = 0.30); I² = 6%									
Test for overall effect: Z = 1.74 (P = 0.08)									
**1.6.2 Disability Immediate Subgroup High Score**									
Haake 2007	−64	21.1	370	−56.3	20.8	361	51.4%	−0.37 [−0.51, −0.22]	2007
Zaringhalam 2010	6.4	2.9	20	8.8	3.8	20	7.8%	−0.70 [−1.34, −0.06]	2010
Moein Jamali Dastjerdi 2024	12.47	11.27	30	20.97	8.62	33	11.3%	−0.84 [−1.36, −0.32]	2024
Lara-Palomo 2024	3.5	2.7	32	5.5	2.7	32	11.7%	−0.73 [−1.24, −0.22]	2024
Subtotal (95% CI)			452			446	82.3%	−0.57 [−0.83, −0.30]	
Heterogeneity: Tau² = 0.03; Chi² = 5.16, df = 3 (P = 0.16); I² = 42%									
Test for overall effect: Z = 4.16 (P < 0.0001)									
Total (95% CI)			497			491	100.0%	−0.49 [−0.68, −0.30]	
Heterogeneity: Tau² = 0.01; Chi² = 6.29, df = 5 (P = 0.28); I² = 20%									
Test for overall effect: Z = 5.11 (P < 0.00001)									
Test for subgroup differences: Chi² = 0.48, df = 1 (P = 0.49), I² = 0%									

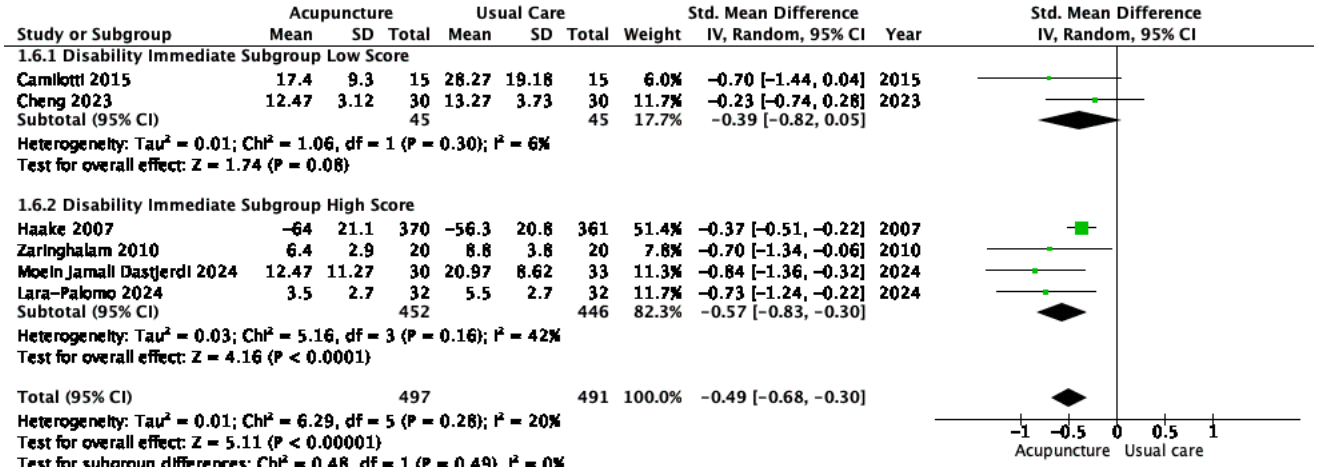

Figure S7. Subgroup analysis for immediate post-treatment disability comparing acupuncture versus usual care, stratified by PEDro score. Trials were grouped based on methodological quality. Significant improvements were observed in the high-quality (PEDro ≥6) subgroup, while results in the low-quality subgroup did not reach statistical significance. No significant interaction was found between subgroups (p = 0.49).

Study or Subgroup	Acupuncture Mean	Acupuncture SD	Acupuncture Total	Usual Care Mean	Usual Care SD	Usual Care Total	Weight	Std. Mean Difference IV, Random, 95% CI	Year
Haake 2007	40.2	22.5	377	52.3	21.2	364	0.0%	−0.55 [−0.70, −0.41]	2007
Zaringhalam 2010	50.1	20.3	20	63.7	24.4	20	32.5%	−0.59 [−1.23, 0.04]	2010
Lara-Palomo 2024	2.8	1.9	32	6.1	1.6	32	33.5%	−1.86 [−2.45, −1.26]	2024
Moein Jamali Dastjerdi 2024	1.93	2.05	30	5.15	1.94	33	34.0%	−1.60 [−2.17, −1.02]	2024
Total (95% CI)			82			85	100.0%	−1.36 [−2.09, −0.63]	
Heterogeneity: Tau² = 0.32; Chi² = 8.92, df = 2 (P = 0.01); I² = 78%									
Test for overall effect: Z = 3.64 (P = 0.0003)									

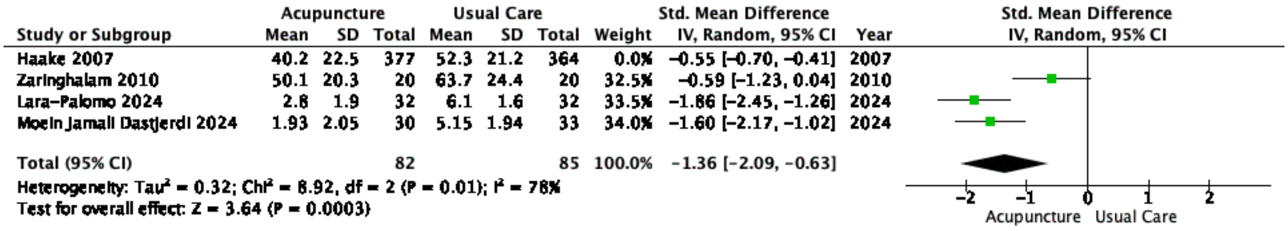

Figure S8. Sensitivity analysis for intermediate-term pain comparing acupuncture versus usual care, excluding Haake 2007.
This analysis excluded the largest trial (Haake 2007) due to atypically small effect size. A strong pooled effect favoring acupuncture was observed (SMD = –1.36; 95% CI –2.09 to –0.63), although heterogeneity remained substantial (I^2 = 78%).

Study or Subgroup	Acupuncture Mean	Acupuncture SD	Acupuncture Total	Usual Care Mean	Usual Care SD	Usual Care Total	Weight	Std. Mean Difference IV, Random, 95% CI	Year
Haake 2007	40.2	22.5	377	52.3	21.2	364	40.2%	−0.55 [−0.70, −0.41]	2007
Zaringhalam 2010	50.1	20.3	20	63.7	24.4	20	29.1%	−0.59 [−1.23, 0.04]	2010
Lara-Palomo 2024	2.8	1.9	32	6.1	1.6	32	0.0%	−1.86 [−2.45, −1.26]	2024
Moein Jamali Dastjerdi 2024	1.93	2.05	30	5.15	1.94	33	30.8%	−1.60 [−2.17, −1.02]	2024
Total (95% CI)			427			417	100.0%	−0.89 [−1.52, −0.25]	
Heterogeneity: Tau² = 0.26; Chi² = 11.99, df = 2 (P = 0.002); I² = 83%									
Test for overall effect: Z = 2.74 (P = 0.006)									

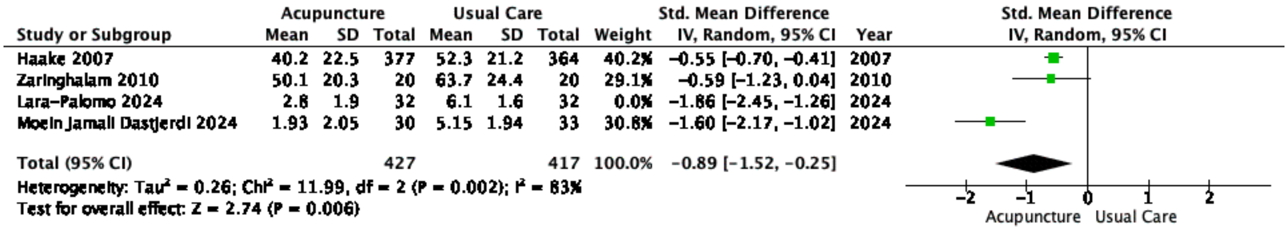

Figure S9. Sensitivity analysis for intermediate-term pain comparing acupuncture versus usual care, excluding Lara-Palomo 2024.
Lara-Palomo 2024 was excluded due to its disproportionately large effect size. The remaining studies showed a statistically significant pooled effect favoring acupuncture (SMD = –0.89; 95% CI –1.52 to –0.25) with persistent heterogeneity (I^2 = 83%).

Study or Subgroup	Acupuncture Mean	Acupuncture SD	Acupuncture Total	Usual Care Mean	Usual Care SD	Usual Care Total	Weight	Std. Mean Difference IV, Random, 95% CI	Year
Haake 2007	40.2	22.5	377	52.3	21.2	364	0.0%	−0.55 [−0.70, −0.41]	2007
Zaringhalam 2010	50.1	20.3	20	63.7	24.4	20	32.5%	−0.59 [−1.23, 0.04]	2010
Lara-Palomo 2024	2.8	1.9	32	6.1	1.6	32	33.5%	−1.86 [−2.45, −1.26]	2024
Moein Jamali Dastjerdi 2024	1.93	2.05	30	5.15	1.94	33	34.0%	−1.60 [−2.17, −1.02]	2024
Total (95% CI)			82			85	100.0%	−1.36 [−2.09, −0.63]	
Heterogeneity: Tau² = 0.32; Chi² = 8.92, df = 2 (P = 0.01); I² = 78%									
Test for overall effect: Z = 3.64 (P = 0.0003)									

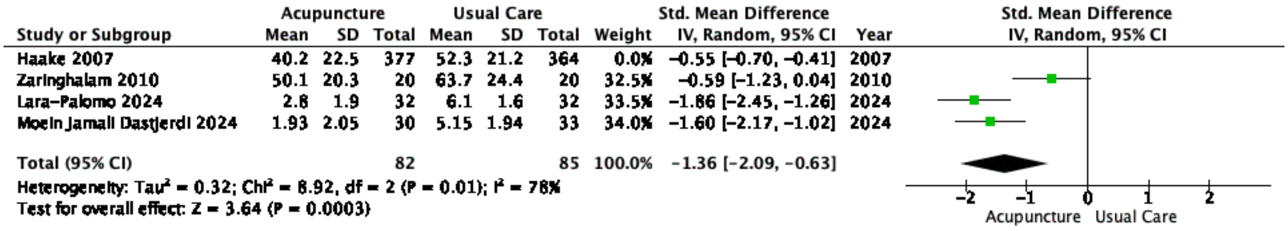

Figure S10. Sensitivity analysis for intermediate-term pain including electroacupuncture studies

Only studies using electroacupuncture were included. Results show a strong effect favoring acupuncture (SMD = –1.36; 95% CI –2.09 to –0.63) with substantial heterogeneity (I^2 = 78%).

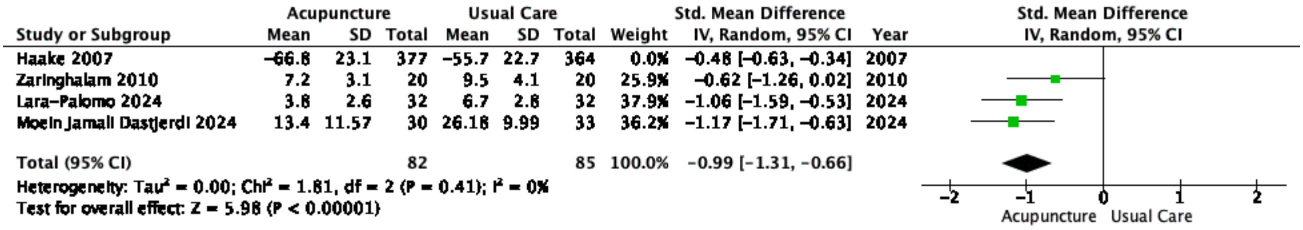

Figure S11. Sensitivity analysis for intermediate-term disability including electroacupuncture studies
This analysis included only trials using electroacupuncture. A large, statistically significant effect favoring acupuncture was observed (SMD = –0.99; 95% CI –1.31 to –0.66) with no observed heterogeneity (I^2 = 0%)

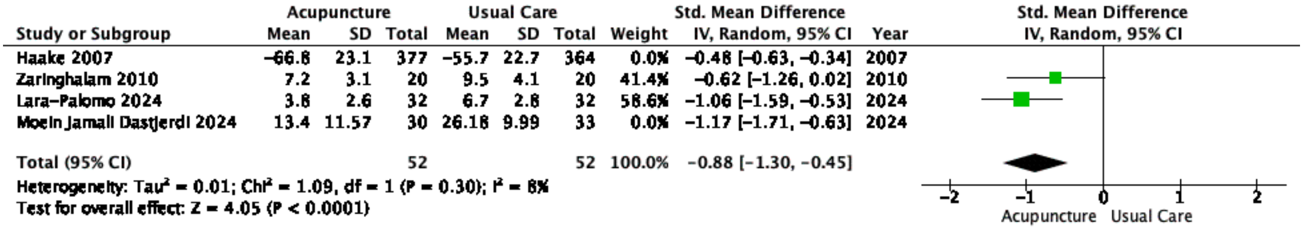

Figure S12. Sensitivity analysis for intermediate-term disability comparing acupuncture versus usual care, excluding Moein Jamali Dastjerdi 2024.
This analysis excluded a statistical outlier with a high effect size and minimal variance. The resulting pooled effect remained significant (SMD = –0.88; 95% CI –1.30 to –0.45) with low heterogeneity (I^2 = 8%).

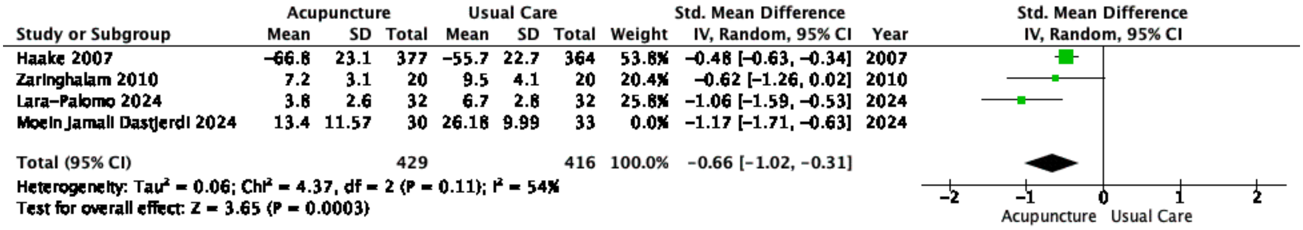

Figure S13. Sensitivity analysis for intermediate-term disability comparing acupuncture versus usual care, including all eligible studies.
All four studies assessing intermediate-term disability were included. Moein Jamali Dastjerdi 2024 contributed no statistical weight. The pooled result (SMD = –0.66; 95% CI –1.02 to –0.31) favored acupuncture with moderate heterogeneity (I^2 = 54%).

Supplementary Material 2: GRADE Summary Tables for Pain and Disability Outcomes

Certainty assessment							№ of patients		Effect		Certainty	Importance
№ of studies	Study design	Risk of bias	Inconsistency	Indirectness	Imprecision	Other considerations	Acupuncture	Usual Care	Relative (95% CI)	Absolute (95% CI)		
Pain												

Certainty assessment							№ of patients		Effect		Certainty	Importance
№ of studies	Study design	Risk of bias	Inconsistency	Indirectness	Imprecision	Other considerations	Acupuncture	Usual Care	Relative (95% CI)	Absolute (95% CI)		
7	randomised trials	serious[a]	not serious	not serious	not serious	publication bias strongly suspected[b]	527	521	-	SMD **0.73 SD lower** (1.04 lower to 0.42 lower)	⊕⊕◯◯ Low[a,b]	IMPORTANT

CI: confidence interval; **SMD:** standardised mean difference

Explanations

a. We downgraded for serious risk of bias because most studies lacked blinding of outcome assessors and did not clearly report allocation concealment.
b. Fewer than 10 studies were included, and several small studies showed large effects. The funnel plot suggested possible asymmetry, and publication bias cannot be ruled out.

Table S1. Immediate Post-Treatment Pain Relief (Primary Outcome)

Seven RCTs (n = 1,048) showed a moderate effect in favor of acupuncture (SMD = –0.73; 95% CI –1.04 to –0.42).
Certainty: Low, downgraded for risk of bias and suspected publication bias

Certainty assessment							№ of patients		Effect		Certainty	Importance
№ of studies	Study design	Risk of bias	Inconsistency	Indirectness	Imprecision	Other considerations	Acupuncture	Usual Care	Relative (95% CI)	Absolute (95% CI)		
**Pain intermediate**												
4	randomised trials	serious[a]	serious[b]	not serious	not serious	publication bias strongly suspected[c]	459	449	-	SMD **1.13 SD lower** (1.82 lower to 0.43 lower)	⊕◯◯◯ Very low[a,b,c]	IMPORTANT

CI: confidence interval; **SMD:** standardised mean difference

Explanations

a. Most included studies did not blind outcome assessors, and one study lacked allocation concealment. Pain is a subjective outcome, and the effect estimate is partially driven by studies at high or unclear risk of bias.
b. Although all studies showed effects favoring acupuncture, heterogeneity was very high (I^2 = 89%), and effect sizes ranged widely. Subgroup analysis helped explain part of the inconsistency
c. Only four studies were included, and three small studies showed large effects. Publication bias cannot be ruled out.

Table S2. Intermediate-Term Pain Relief (Primary Outcome)

Four RCTs (n = 908) showed a large effect favoring acupuncture (SMD = –1.13; 95% CI –1.82 to –0.43).
Certainty: Very Low, due to risk of bias, inconsistency, and suspected publication bias.

Certainty assessment							№ of patients		Effect		Certainty	Importance
№ of studies	Study design	Risk of bias	Inconsistency	Indirectness	Imprecision	Other considerations	Acupuncture	Usual Care	Relative (95% CI)	Absolute (95% CI)		
**Disability Immediate**												
6	randomised trials	serious[a]	not serious	not serious	not serious	publication bias strongly suspected[b]	497	491	-	SMD **0.49 SD lower** (0.68 lower to 0.3 lower)	⊕⊕◯◯ Low[a,b]	IMPORTANT

CI: confidence interval; **SMD:** standardised mean difference

Explanations

a. Most studies had unclear allocation concealment or lacked assessor blinding, which is important for a subjective outcome like disability.
b. Fewer than 10 studies were included, and several small studies showed large effects. The funnel plot suggested possible asymmetry, and publication bias cannot be ruled out.

Table S3. Immediate Post-Treatment Disability Reduction (Secondary Outcome)

Six RCTs (n = 988) showed a small-to-moderate effect (SMD = –0.49; 95% CI –0.68 to –0.30). **Certainty: Low**, downgraded for risk of bias and suspected publication bias

Certainty assessment							№ of patients		Effect		Certainty	Importance
№ of studies	Study design	Risk of bias	Inconsistency	Indirectness	Imprecision	Other considerations	Acupuncture	Usual Care	Relative (95% CI)	Absolute (95% CI)		
**Disability Intermediate**												
4	randomised trials	serious[a]	serious[b]	not serious	serious[c]	publication bias strongly suspected[d]	459	449	-	SMD **0.79 SD lower** (1.18 lower to 0.41 lower)	⊕◯◯◯ Very low[a,b,c,d]	

CI: confidence interval; **SMD:** standardised mean difference

Explanations

a. Most studies lacked assessor blinding for a subjective outcome like disability. One study also lacked allocation concealment. We downgraded for serious risk of bias.
b. All studies showed effects in the same direction, but heterogeneity was substantial (I^2 = 68%) and the magnitude of effects varied. Subgroup and sensitivity analyses suggest the effect is robust, but not all inconsistency was explained.
c. The confidence interval did not cross the null but ranged from moderate to borderline-small effects (–1.18 to –0.41). Total sample size was near threshold, and the estimate was influenced by small studies with large effects. We downgraded for serious imprecision.
d. Fewer than 10 studies were included, and several small studies showed large effects. The largest study had the smallest effect size, suggesting potential publication bias.

Table S4. Intermediate-Term Disability Reduction (Secondary Outcome)

Four RCTs (n = 908) showed a moderate-to-large effect (SMD = –0.79; 95% CI –1.18 to –0.41). **Certainty: Very Low**, downgraded due to risk of bias, inconsistency, imprecision, and suspected publication bias

Supplementary Material 3: PEDro Scores by Study and Methodological Domain

Study	Eligibility Criteria (Not Scored)	Random Allocation	Concealed Allocation	Baseline Similarity	Blinding of Subjects	Blinding of Therapists	Blinding of Assessors	>85% Follow-Up	ITT Analysis	Between-Group Comparisons	Point Measures & Variability	Total Score
Haake et Al 2007	YES	YES	YES	YES	NO	NO	YES	YES	YES	YES	YES	8/10
Zaringhalam et al 2010	YES	YES	YES	YES	NO	NO	NO	YES	NO	YES	YES	6/10
Shankar et al 2011	YES	YES	NO	YES	NO	NO	NO	YES	NO	YES	YES	5/10
Camilotti et al 2015	YES	YES	NO	YES	NO	NO	NO	NO	NO	YES	YES	4/10
Meng et al 2022	YES	YES	YES	YES	NO	NO	YES	YES	YES	YES	YES	8/10
Cheng et al 2023	YES	YES	NO	YES	NO	NO	NO	YES	NO	YES	YES	5/10
Lara – Palomo et al 2024	YES	YES	YES	YES	NO	NO	YES	YES	YES	YES	YES	8/10
Moein Jamali Dastjerdi et al 2024	YES	YES	NO	YES	NO	NO	YES	YES	YES	YES	YES	7/10

Table S5: This table presents PEDro scores for each included randomized controlled trial assessing acupuncture versus usual care in chronic low back pain. Each study was assessed across 11 PEDro methodological domains (with eligibility criteria not contributing to the total score). Scores reflect trial quality, with higher scores indicating lower risk of bias. The most frequent limitations across studies were lack of blinding of participants, therapists, and assessors.

Supplementary Material 4: Funnel Plots for Publication Bias Assessment

Funnel plots for each meta-analysis of pain and disability outcomes are presented. Funnel plots visually assess potential publication
bias based on the distribution of effect sizes (SMD) relative to their standard errors. Asymmetry suggests small-study effects or
reporting bias.

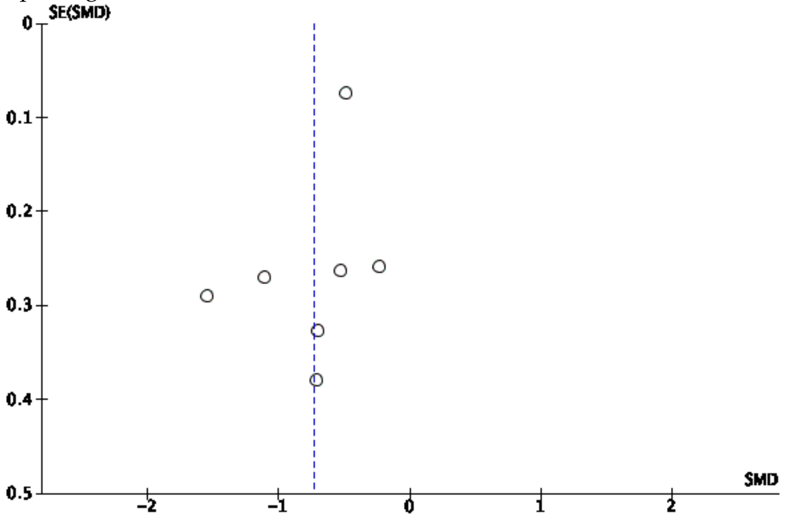

Figure S14. Funnel Plot for Immediate Post-Treatment Pain (Primary Outcome)
Funnel plot of seven trials assessing immediate post-treatment pain relief comparing acupuncture to usual care. Visual inspection
shows asymmetry, with small studies reporting larger effects, suggesting possible publication bias.

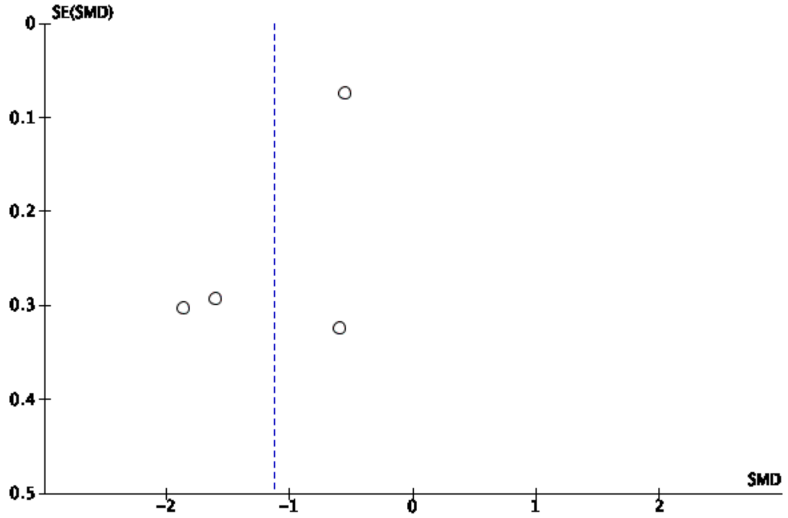

Figure S15. Funnel Plot for Intermediate-Term Pain Relief (Primary Outcome)
Funnel plot of four trials assessing intermediate-term pain relief. The plot shows marked asymmetry, suggesting potential publication bias and small-study effects.

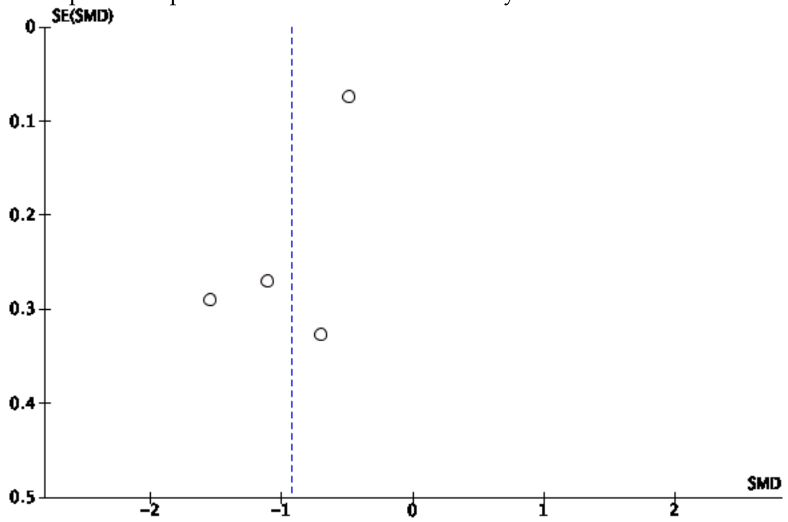

Figure S16. Funnel Plot for Immediate Post-Treatment Pain (Studies with PEDro >6)
Funnel plot of five trials with PEDro scores >6 assessing immediate post-treatment pain. Slight asymmetry is observed, but limited number of studies reduces interpretability regarding publication bias.

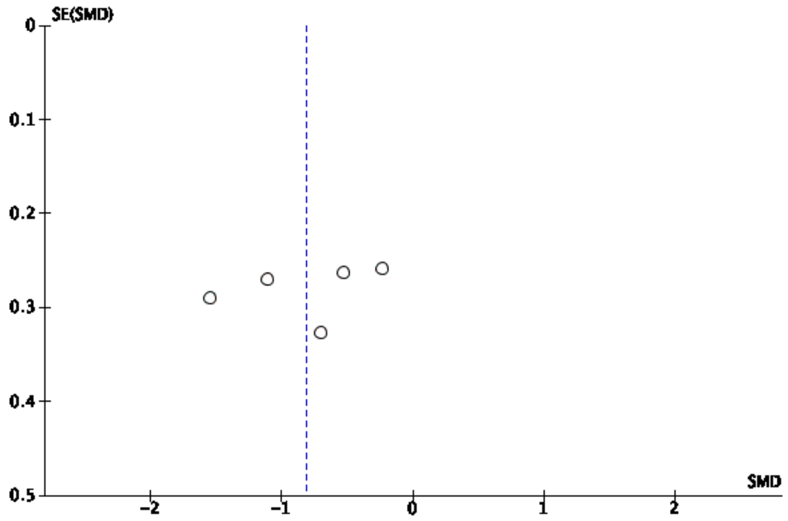

Figure S17. Funnel Plot for Immediate Post-Treatment Pain (Electroacupuncture Only)

Funnel plot of five electroacupuncture trials evaluating immediate post-treatment pain relief. The distribution appears relatively symmetric, suggesting a lower likelihood of publication bias within this subgroup.

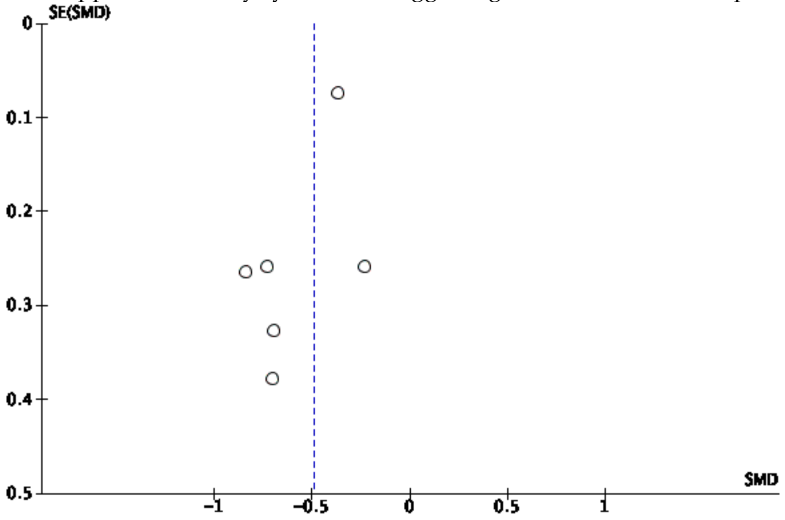

Figure S18. Funnel Plot for Immediate Post-Treatment Disability

Funnel plot of six studies assessing disability immediately post-treatment. Mild asymmetry is observed; however, with few studies, firm conclusions about publication bias remain uncertain.

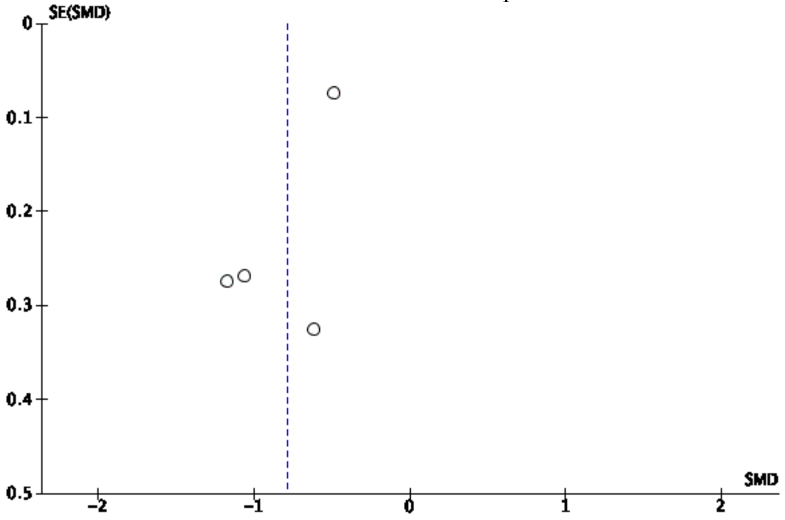

Figure S19. Funnel Plot for Intermediate-Term Disability

Funnel plot of four studies reporting on disability at intermediate-term follow-up. The distribution suggests slight asymmetry, but due to the limited number of studies, assessment of small-study effects remains inconclusive.

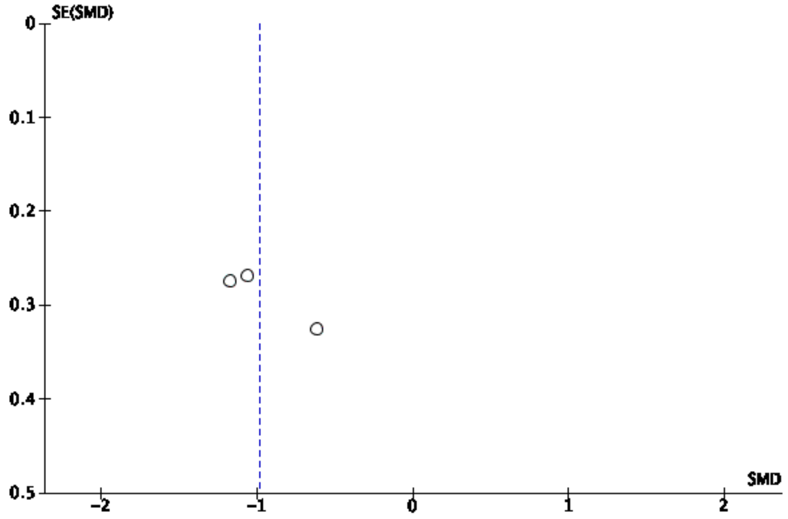

Figure S20. Funnel Plot for Intermediate-Term Disability (EA Only Studies)

Funnel plot of electroacupuncture-only studies reporting on intermediate-term disability outcomes. The plot displays limited dispersion and symmetry; however, the small number of studies restricts formal evaluation of publication bias.

Supplementary Material 5: PRISMA 2020 CHECKLIST

Section and Topic	Item #	Checklist item	Location where item is reported
**TITLE**			
Title	1	Identify the report as a systematic review.	p.1
**ABSTRACT**			
Abstract	2	See the PRISMA 2020 for Abstracts checklist.	p.1
**INTRODUCTION**			
Rationale	3	Describe the rationale for the review in the context of existing knowledge.	p.1,2
Objectives	4	Provide an explicit statement of the objective(s) or question(s) the review addresses.	p.2
**METHODS**			
Eligibility criteria	5	Specify the inclusion and exclusion criteria for the review and how studies were grouped for the syntheses.	p.3
Information sources	6	Specify all databases, registers, websites, organisations, reference lists and other sources searched or consulted to identify studies. Specify the date when each source was last searched or consulted.	p.3
Search strategy	7	Present the full search strategies for all databases, registers and websites, including any filters and limits used.	p.3
Selection process	8	Specify the methods used to decide whether a study met the inclusion criteria of the review, including how many reviewers screened each record and each report retrieved, whether they worked independently, and if applicable, details of automation tools used in the process.	p.3
Data collection process	9	Specify the methods used to collect data from reports, including how many reviewers collected data from each report, whether they worked independently, any processes for obtaining or confirming data from study investigators, and if applicable, details of automation tools used in the process.	p.3
Data items	10a	List and define all outcomes for which data were sought. Specify whether all results that were compatible with each outcome domain in each study were sought (e.g. for all measures, time points, analyses), and if not, the methods used to decide which results to collect.	p.3,4
	10b	List and define all other variables for which data were sought (e.g. participant and intervention characteristics, funding sources). Describe any assumptions made about any missing or unclear information.	p.3,4
Study risk of bias assessment	11	Specify the methods used to assess risk of bias in the included studies, including details of the tool(s) used, how many reviewers assessed each study and whether they worked independently, and if applicable, details of automation tools used in the process.	p.3,4
Effect measures	12	Specify for each outcome the effect measure(s) (e.g. risk ratio, mean difference) used in the synthesis or presentation of results.	p.4
Synthesis methods	13a	Describe the processes used to decide which studies were eligible for each synthesis (e.g. tabulating the study intervention characteristics and comparing against the planned groups for each synthesis (item #5)).	p.4
	13b	Describe any methods required to prepare the data for presentation or synthesis, such as handling of missing summary statistics, or data conversions.	p.3,4
	13c	Describe any methods used to tabulate or visually display results of individual studies and syntheses.	p.3,4, supplementary

Section and Topic	Item #	Checklist item	Location where item is reported
	13d	Describe any methods used to synthesize results and provide a rationale for the choice(s). If meta-analysis was performed, describe the model(s), method(s) to identify the presence and extent of statistical heterogeneity, and software package(s) used.	p.4
	13e	Describe any methods used to explore possible causes of heterogeneity among study results (e.g. subgroup analysis, meta-regression).	p.4
	13f	Describe any sensitivity analyses conducted to assess robustness of the synthesized results.	p.4
Reporting bias assessment	14	Describe any methods used to assess risk of bias due to missing results in a synthesis (arising from reporting biases).	p.4
Certainty assessment	15	Describe any methods used to assess certainty (or confidence) in the body of evidence for an outcome.	p.4
**RESULTS**			
Study selection	16a	Describe the results of the search and selection process, from the number of records identified in the search to the number of studies included in the review, ideally using a flow diagram.	p.4,5
	16b	Cite studies that might appear to meet the inclusion criteria, but which were excluded, and explain why they were excluded.	
Study characteristics	17	Cite each included study and present its characteristics.	p.6
Risk of bias in studies	18	Present assessments of risk of bias for each included study.	Sup.3
Results of individual studies	19	For all outcomes, present, for each study: (a) summary statistics for each group (where appropriate) and (b) an effect estimate and its precision (e.g. confidence/credible interval), ideally using structured tables or plots.	p.6
Results of syntheses	20a	For each synthesis, briefly summarise the characteristics and risk of bias among contributing studies.	p.8-11
	20b	Present results of all statistical syntheses conducted. If meta-analysis was done, present for each the summary estimate and its precision (e.g. confidence/credible interval) and measures of statistical heterogeneity. If comparing groups, describe the direction of the effect.	p.8-11
	20c	Present results of all investigations of possible causes of heterogeneity among study results.	p.8-11
	20d	Present results of all sensitivity analyses conducted to assess the robustness of the synthesized results.	p.8-11
Reporting biases	21	Present assessments of risk of bias due to missing results (arising from reporting biases) for each synthesis assessed.	p.10
Certainty of evidence	22	Present assessments of certainty (or confidence) in the body of evidence for each outcome assessed.	p.10,11
**DISCUSSION**			
Discussion	23a	Provide a general interpretation of the results in the context of other evidence.	p.11-13
	23b	Discuss any limitations of the evidence included in the review.	p.12-13
	23c	Discuss any limitations of the review processes used.	p.12-13
	23d	Discuss implications of the results for practice, policy, and future research.	p.13

Section and Topic	Item #	Checklist item	Location where item is reported
**OTHER INFORMATION**			
Registration and protocol	24a	Provide registration information for the review, including register name and registration number, or state that the review was not registered.	p.3
	24b	Indicate where the review protocol can be accessed, or state that a protocol was not prepared.	p.3
	24c	Describe and explain any amendments to information provided at registration or in the protocol.	p.4
Support	25	Describe sources of financial or non-financial support for the review, and the role of the funders or sponsors in the review.	p.13
Competing interests	26	Declare any competing interests of review authors.	p.13
Availability of data, code and other materials	27	Report which of the following are publicly available and where they can be found: template data collection forms; data extracted from included studies; data used for all analyses; analytic code; any other materials used in the review.	p.13
